# Anemia at the Initiation of Tuberculosis Therapy Is Associated with Delayed Sputum Conversion among Pulmonary Tuberculosis Patients in Dar-es-Salaam, Tanzania

**DOI:** 10.1371/journal.pone.0091229

**Published:** 2014-03-18

**Authors:** Tumaini J. Nagu, Donna Spiegelman, Ellen Hertzmark, Said Aboud, Julie Makani, Mecky I. Matee, Wafaie Fawzi, Ferdinand Mugusi

**Affiliations:** 1 Department of Internal Medicine, Muhimbili University of Health and Allied Sciences (MUHAS), Dar- es- Salaam, Tanzania; 2 Department of Laboratory Medicine, Karolinska Institutet (KI), Stockholm, Sweden; 3 Department of Epidemiology, Harvard School of Public Health (HSPH), Boston, Massachusetts, United States of America; 4 Department of Biostatistics, Harvard School of Public Health (HSPH), Boston, Massachusetts, United States of America; 5 Department of Microbiology and Immunology, Muhimbili University of Health and Allied Sciences (MUHAS), Dar es Salaam, Tanzania; 6 Department of Hematology, Muhimbili University of Health and Allied Sciences (MUHAS), Dar- es- Salaam, Tanzania; 7 Department of Global Health and Population, Harvard School of Public Health (HSPH), Boston, Massachusetts, United States of America; Temple University School of Medicine, United States of America

## Abstract

**Background:**

Pulmonary tuberculosis and anemia are both prevalent in Tanzania. There is limited and inconsistent literature on the association between anemia and sputum conversion following tuberculosis treatment.

**Methods:**

Newly diagnosed sputum smear positive pulmonary tuberculosis patients aged ≥15 years initiating on standard anti tuberculosis therapy were recruited from 14 of 54 tuberculosis clinics in Dar es Salaam. Patients were receiving medication according to the recommended short course Directly Observed Therapy (DOT) strategy and were followed up prospectively until completion of treatment (six months). Patients were evaluated before initiation of TB treatment by performing the following; clinical history, physical examination, complete blood counts, serum biochemistry and sputum microscopy. Sputum smears were re-examined at two months of anti-tuberculosis therapy for presence of acid fast bacilli. Anemia was defined as hemoglobin <13 g/dl (males) or <12 g/dl (females). Log-binomial regression was used to assess the association between anemia and sputum conversion at two months.

**Results:**

Of the 1245 patients included in the study, 86% were anemic and 7% were sputum smear positive at two months of anti-tuberculosis therapy. Anemic patients were three times more likely to have sputum positive smear as compared to non-anemic patients at two months (RR = 3.05; 95% CI 1.11–8.40) p = 0.03. The risk for sputum positive smear results increased with severity of anemia (P for trend <0.01).

**Conclusion:**

Baseline anemia is associated with increased risk for persistent positive sputum smears at two months of tuberculosis treatment. Future studies should evaluate the mechanisms for TB-associated anemia as well as the role of intervention for anemia among TB patients.

## Introduction

Tuberculosis (TB) is the world's second most common cause of death from infectious diseases [1], [2]. In 2011, a total of 8.7 million new active TB cases and 1.4 million TB related deaths were estimated worldwide; 70% of these deaths were among HIV uninfected people [2]. It is evident there is need to strengthen strategies for infection control, identification and management of TB patients at increased risk for death in addition to identifying effective chemotherapy, in order to reduce TB related morbidity and mortality.

Smear positivity is the most important predictor of TB infectiousness [3], [4]. When smear positive TB patients initiate TB therapy containing rifampicin and isoniazid, there is a rapid multifold reduction in bacillary load expelled in sputum which minimizes the risk for transmission [5]. About 90% of patients are likely to become smear and/or culture negative (smear and/or culture conversion) after two to three months of TB chemotherapy [6]–[9]. However, approximately 10% of TB patients are still culture positive 60 days following initiation of anti TB therapy [6], implying a potential for persistent infectiousness. Furthermore, longer smear conversion times have been associated with subsequent poor TB treatment outcomes and relapse within two years of follow up [10], [11]. Some risk factors have been identified for delayed time to smear conversion. These include cavitatory lesions, high initial sputum smear acid fast bacillary (AFB) counts, multi drug resistant TB, old age, diabetes and duration of symptoms before treatment [10], [12], [13].

Anemia is estimated to occur in 1–8% of the world's population [14]. It is the third cause of global years lived with disability (YLD), responsible for more than 600 YLD per 100,000 people [14], and shows regional variation in incidence and prevalence [14], [15]. In eastern sub-Saharan Africa, anemia is the leading cause of years lived with disability (YLDs) [14]. The prevalence of anemia among TB patients ranges between 30–94% [16]–[20]. It has been shown that anemia is more likely to occur among TB patients compared to healthy controls [21]. More importantly, anemia is associated with more severe forms of TB [22] and poorer TB outcomes, including deaths [17], [19], [23]. There is scanty and inconsistent literature on the relationship between anemia and sputum smear conversion. One study showed no association between anemia and TB culture positivity at one month of TB therapy [17], while another found a higher mean hemoglobin following sputum smear conversion at two months of TB therapy compared to baseline hemoglobin [22].

We conducted a prospective study to examine the effect of baseline anemia on sputum smear conversion at two months of anti-tuberculosis chemotherapy.

## Materials and Methods

### Ethics statement

The study was cleared by Muhimbili University of Health and Allied Sciences (MUHAS) ethical review board. Permission to conduct the study was obtained from the Kinondoni, Temeke and Ilala Municipal directors for health. Prior informed written consent was sought from all participating patients. Patients aged 15–17 years gave an assent to participate in the study, and their parents or guardians signed a written consent. All patients were managed according to the national TB management protocol.

### Setting and study design

This prospective study was conducted in Dar es Salaam, Tanzania, a commercial city with about 4 million inhabitants and a population density of 3133 per square kilometer, compared to the Tanzanian national average of 51 inhabitants per square kilometer [24]. Dar es Salaam had the highest TB notification rate, with 22% of 61,838 TB cases reported in Tanzania in 2011 [25]. This study was conducted in between October 28^th^ 2010 and December 20^th^ 2011 in the 14 largest TB clinics (of the total 54 TB diagnostic and treatment clinics in Dar es Salaam), based on the National Tuberculosis data. The list resulted in 4 or 5 clinics from each of the three municipalities in Dar es Salaam.

### Patients

Study participants were consenting pulmonary tuberculosis (PTB) patients (defined as having at least two positive results of smear for acid fast bacilli (AFB) on microscopy), aged 15 years and above. Patients were treated according to the national guidelines for the management of TB [26], which stipulates daily direct observed therapy (DOT) throughout the six months of anti-TB drug administration. In Tanzania, patients have the choice of taking medication at Health facility (facility DOT) or at their home in the presence of a community treatment supporter (community DOT). For community DOT, a weekly supply of TB drugs is provided, while ensuring adherence during refill using patients' treatment cards. Therapy consisted of a two month intensive phase of daily rifampicin, isoniazid, pyrazinamide and ethambutol. At the end of the intensive phase, PTB patients whose follow up smears were negative for AFB on microscopy continued with daily rifampicin and isoniazid for an additional four months. Patients whose two months follow up sputum smears were positive for AFB on microscopy had an extended intensive phase for one more month. We excluded all patients who had used anti TB drugs within the past two years or who stated that they did not intend to stay in Dar es Salaam until completion of their treatment.

### Study procedures

Following consent, clinicians conducted interviews, performed physical examinations, filled the case report forms (CRF) and obtain sputum and blood samples. Patients were reviewed at two and five and six months by attending clinicians who also recorded the sputum smear results and patient outcomes at the end of treatment as stipulated by the National TB and Leprosy Control Programme [26].

### Laboratory investigations

Sputum smears were prepared using Ziehl–Neelsen (ZN) stain and were examined for acid fast bacilli (AFB) at: baseline, and at two and five months of TB treatment. Results were reported on the laboratory request forms and documented on the clinic TB registers. Drug susceptibility testing (DST) was performed at the central reference Tuberculosis Laboratory located at Muhimbili National Hospital. We initially performed DST using the BACTEC Mycobacteria Growth Indicator Tube (MGIT) – (Beckton-Dickinson) technique according to manufacturer's instructions. Due to limited resources, we had to change to conventional, egg-based Lowenstein Jensen (LJ) media. Each sample was then cultured in Lowenstein Jensen media at 37°C for up to 8 weeks. Plates were examined weekly for growth. Colonies were identified according to criteria based on the speed of growth and macroscopic features e.g. roughness and pigment production and ZN smear microscopy. DST was performed by the proportion method. Complete blood count (hemoglobin; white cell count, neutrophils, lymphocytes, monocytes, eosinophil and basophil; and platelets) was performed using the ACT5 DIFF hematology analyser (Beckman Coulter, Miami, Florida). Clinical chemistry: Alanine aminotransferase, (ALT), bilirubin, serum creatinine, urea and albumin were performed using Cobas integra 400 plus Chemistry Analyzer (Roche, Rotkreuz, Switzerland). HIV infection was determined according to the National HIV screening algorithm. The algorithm requires serial testing using Determine™ HIV-1/2 (Inverness Medical Japan Co. Ltd, Japan) then Uni-Gold™ HIV-1/2 (Trinity Biotech, Wicklow, Ireland). Enzyme Linked Immunosorbent Assay (ELISA) method was used as a tie breaker in discordant rapid test results.

### Quality assurance

Quality assurance was accomplished by assessing the quality and adequacy of specimens and by monitoring microscopy and culture procedures according to the established laboratory operating procedures. Patients were requested to provide additional specimens in case of submitting either inadequate amounts or salivary samples. For smear microscopy, positive and negative control slides were included with each batch of new reagents in a blind manner. Random sputum specimens were reviewed at CTRL as quality assurance measure. Additional quality measures for culture included monitoring of quality of water, decontamination, digestion and concentration procedures, inspissations and incubation temperatures, and measurement and adjustment of pH of culture media. A standard laboratory strain M. tuberculosis H37Rv was used as a positive control.

### Data management and statistical analysis

Information from CRFs was double entered by trained data clerks using Epi6 statistical software. Statistical analysis was conducted using SAS version 9.3 (SAS Institute, Cary, NC) statistical software. We used proportions to describe the basic characteristics of the study population at the time of enrolment. Log-binomial regression was used to assess the association of baseline anemia with delayed sputum smear conversion [27], [28]. When log-binomial models did not converge, the Poisson approximation was used. Univariate and multivariate analyses were performed. Variables with p≤0.2 in univariate analysis were included [29] in the multivariate model, in addition to HIV infection and body mass index (BMI).

The outcome measure for this study was delayed sputum smear conversion, defined as sputum smear positive for AFB at two months of antiTB therapy. The primary exposure of interest was anemia at baseline, defined per World Health Organization (WHO) guidelines as hemoglobin <13 g/dl (males) or hemoglobin <12 g/dl (female) [30]. Anemia was further categorized according to severity with the following hemoglobin cut off points: mild anemia; 11.00 – <13 g/dl (male) and 11.00 – <12 g/dl (female); moderate anemia 8.00 – <11 (both sexes); severe anemia hemoglobin less than 8 g/dl for both sexes [30].

The primary multivariate analysis for delayed sputum smear conversion was performed with anemia as a binary variable (Yes or No). In secondary analysis, we used the anemia severity variable (no anemia, mild, moderate, severe). Both models adjusted for the same covariates. In the multivariate model, we adjusted for potential confounding effects of age (15–29 years, 30–50 years, >50 years), sex (male/female), HIV status (positive/negative), cigarette smoking (never, past or current) ,alcohol consumption (never, past or current), substance abuse (never, past or current), body mass index (BMI) categories (<18.5 kg/m^2^,18.5–25 kg/m^2^, >25 kg/m^2^), duration of illness(<4 weeks vs. ≥4 weeks or continuous variable with spline term), lymphocyte count (<1.0×10^6^cell/µl vs ≥1.0×10^6^cells/µl) platelet counts (<150,000cells/µl, 150,000–400,000 cells/µl or >400,000 cells/µl) and resistance to rifampicin and/or isoniazid (yes/no). In the multivariate models missing indicators were used for covariates with large numbers of missing values. The relation between hemoglobin concentration and the risk of delayed sputum conversion was examined for nonlinearity using restricted cubic splines [31]–[32], adjusted for the covariates as described for our multivariate model. We also modeled our outcomes omitting platelet count and HIV status, which could mediate the effect of anemia on delayed sputum conversion. P<0.05 was considered significant.

## Results

During the study period October 28^th^ 2010 and December 20^th^ 2011, a total of 1805 sputum smear positive patients were enrolled and studied. Among these, 357 (19.8%) patients were missing sputum smear results at two months and a further 203 (11.2%) patients were missing baseline hemoglobin. We therefore included 1245 patients in this study with baseline hemoglobin and two months sputum smear results available.

Patients who were excluded were similar to the included patients in: demographic characteristics (age, sex, marital and education status); their economic status (monthly income and expenditure on food); cigarette smoking and alcohol status; disease severity (BMI and duration of symptoms); proportion with HIV infection and proportion with rifampicin and/or isoniazid resistance. Excluded patients had significantly lower lymphocyte counts (median 1.4×10^6^ vs. 1.5×10^6^ cells/µl) p = 0.05 and were more likely to have a history of current (3.2 vs. 1.9) or past (7.29 vs. 2.9) substance abuse (p = <0.0001).

The median age (IQR) of the participants was 32 (25–40) years. As shown in Table 1, most participants (67%) were males. The median monthly income and daily expenditure on food for the study participants were 100 USD and 3 USD respectively. About a third, 359 (30%) of the study participants had HIV infection, 3 (0.46%) had rifampicin resistance, of whom one had multi drug resistant (MDR) TB, and 20 patients (3.05%), had resistance to either rifampicin or isoniazid.

**Table 1:** Baseline characteristics of patients at the time of initiation of Tuberculosis (TB) therapy (N = 1245).

Variable	Missing No (%)	No	%
**Male sex**	0	831	67
**Age (years)**	32 (2.57)		
15 - <30		485	40
30–50		618	51
>50		110	9
**Marital Status**	16 (1.29)		
Never married		580	47
Cohabiting/married		531	43
Divorced/widowed		118	10
**Education status**	20 (1.61)		
No formal education		77	6
Primary school		881	72
Secondary and above		267	22
**Monthly income (USD)**	275 (22.09)		
<100		418	43
≥100		552	57
**Daily family expenditure on food (USD)**	200 (16.06)		
<3		432	41
≥3		613	59
**Smoking**	13 (1.04)		
Never		912	74
Quit		263	21
Current		57	5
**Alcohol**	20 (1.61)		
Never		779	63
Quit		352	29
Current		94	8
**Substance abuse**	35 (2.81)		
Never		1154	95
Quit		34	3
Current		22	2
**BMI categories kg/m^2^**	126 (9.96)		
<18.5		575	51
18.5 - <25		501	45
≥25		45	4
**Duration of illness (weeks)**	103 (8.2)		
<4		266	23
≥4		876	77
**HIV positive**	58 (4.66)	359	30
**Resistance to anti TB drugs**			
Isoniazid (H) resistance	590 (47.45)	18	2.75
Rifampicin (R) resistance	590 (47.45)	3	0.46
R or H resistance	590 (47.45)	20	3.05
**Lymphocyte count (×10^6^ cells/µl)**	2 (0.2)		
<1.0		302	24
≥1.0		941	76
**MCV**	3 (0.24)		
<80		615	50
≥80		627	50
**Platelets (cells/µl)**	4 (0.3)		
<150,000		123	10
150,000–400,000		658	53
>400,000		460	37
**Serum creatinine**	221 (17.75)		
≤110		961	94
>110		63	6

Anemia was present in 1067 (86%) patients of the study population, of the anemic patients two thirds 697 (65%) had moderate to severe anemia. Delayed sputum smear conversion occurred in 82 (7%) patients.

Table 2 shows the association between anemia at the time of enrollment and delayed sputum smear conversion at two months of TB therapy. In the adjusted model, patients with anemia at the time of enrolment were three times more likely to have delayed sputum smear conversion than patients without anemia, (RR = 3.05; 95% CI 1.11–8.40; p = 0.03) (Table 2). When anemia severity was modeled in a separate multivariable analysis, the likelihood of delayed sputum smear conversion increased with severity of anemia (trend test P<0.01). Patients with mild anemia had a nearly 3-fold increased risk of delayed sputum smear conversion (RR = 2.72; 95%CI 0.94–7.85) compared to those without anemia, whereas patients with severe anemia were five times as likely to have delayed sputum smear conversion as those without anemia (RR = 5.08; 95% CI 1.65–15.62) p<0.01.

**Table 2:** The association between delayed sputum conversion and anemia among Tuberculosis patients (N = 1245)*.

Predictor	Event/# at risk	Unadjusted model		Adjusted model	
		RR (95% CI)	P	RR (95% CI)	P
**Anemia**					
No anemia	4/178	1		1	
Anemia	78/1067	3.25 (1.21–8.77)	0.02	3.05 (1.11–8.40)	0.03
**Anemia severity**					
No	4/178	1		1	
Mild	24/370	2.89 (1.02–8.19)		2.72 (0.94–7.85)	
Moderate	38/542	3.12 (1.13–8.62)		3. 02 (1.07–8.55)	
Severe	16/155	4.59 (1.57–13.45)	<0.01	5.08 (1.65–15.62)	<0.01**
**Hemoglobin (g/dl)**	82/1245	0.87 (0.80–0.95)	<0.01	0.84 (0.76–0.94)	**<**0.01

*Two separate models for (anemia and anemia severity) were each adjusted for: age, sex, HIV status, smoking, alcohol, drug abuse, body mass index (BMI), duration of illness, lymphocytes, platelets count and resistance to rifampicin and/or Isoniazid.

**Trend test p-value.

Examination of the relation of baseline hemoglobin to delayed sputum conversion showed a linear relation, with and adjusted risk ratio of 0.84 (95% CI 0.76–0.94; p<0.01) per gram of hemoglobin per deciliter. No evidence of nonlinearity was found. Modification of the basic models, either by using continuous duration of illness with the spline variable or by excluding platelet count and HIV status, did not materially change the results. The relative risks for anemia with or without platelet count and HIV status ranged from 2.86 to 3.12 respectively.

Other potential predictors of delayed sputum smear conversion that were investigated in this study are shown in Table 3. Older patients aged 30–50 years had a higher risk for delayed sputum smear conversion (RR = 1.93; 95% CI 1.13–3.31) compared to patients aged 15 – <30 years. Current smokers were about twice as likely to have delayed sputum smear conversion (RR 2.13; 95%CI 1.02–4.45) as never smokers (p = 0.04). Resistance to either rifampicin or isoniazid was significantly associated with delayed sputum smear conversion (RR = 2.74; 95% CI 1.26–5.97) p = 0.01. Patients with low lymphocyte count (<1.0×10^6^ cells/µl) were significantly more likely to delay sputum smear conversion (RR = 1.77; 95% CI 1.16–2.70) compared to those with lymphocyte count of 1.0×10^6^ cells/µl or above (p = 0.008). We found no evidence for increased risk for delayed sputum smear conversion among those who had quit smoking. There was no association between delayed sputum smear conversion and BMI or HIV infection. Alcohol use, substance abuse, and duration of illness were significantly associated with delayed sputum smear conversion at univariate but not multivariate analysis (Table 3). Sex, monthly income, daily expenditure on food, marital and education status were not significantly associated with delayed sputum smear conversion (Table 3).

**Table 3:** Predictors of delayed sputum smear conversion among Tuberculosis patients (n = 1245)*.

	Univariate analysis		Multivariate analysis	
Predictor	RR (95% CI)	P	RR (95% CI)	P
**Anemia**				
No	1		1	
Yes	3.25 (1.21–8.77)	0.02	3.05 (1.11–8.40)	0.03
**Age (Years)**				
15 – <30	1		1	
30–50	2.18 (1.31–3.65)		1.93 (1.13–3.31)	
>50	1.86 (0.83–4.13)	<0.01	1.88 (0.85–4.14)	0.03
**Sex**				
Female	1		1	
Male	1.96 (1.13–3.21)	0.02	1.36 (0.76–2.43)	0.30
**Smoking status**				
Never	1		1	
Quit	1.95 (1.20–3.12)		1.35 (0.76–2.42)	
Current	4.33 (2.43–7.12)	<.0001	2.13 (1.02–4.45)	0.04
**Alcohol**				
Never	1		1	
Quit	1.55 (0.90–2.47)		1.05 (0.60–1.82)	
Current	2.79 (1.50–4.84)	0.001	1.21 (0.59–2.50)	0.68
**Substance abuse**				
Never	1		1	
Quit	1.92 (0.74–4.94)		1.23 (0.53–2.85)	
Current	3.70 (1.66–8.25)	<0.001	2.12 (0.95–4.73)	0.07
**BMI categories (Kg/m^3^)**				
<18.50	0.87 (0.56–1.35)		0.72 (0.48–1.10)	
18.5–24.99	1	0.91	1	0.63
≥25.00	0.03 (0.04–2.14)		0.31(0.04–2.23)	
**Duration of illness (weeks)**				
≥4	1		1	
<4	2.24 (1.25–3.99)	<0.01	1.48 (0.80–2.72)	0.21
**Rifampicin/Isoniazid Resistance**				
No	1		1	
Yes	2.33 (0.80–6.75)	0.12	2.74 (1.26–5.97)	0.01
**HIV infection**				
No	1		1	
Yes	0.85 (0.52–1.38)	0.52	0.77 (0.46–1.29)	0.32
**Lymphocytes ×10^6^ (cells/µl)**				
≥1.0	1		1	
<1.0	1.80 (1.17–2.77)	<0.01	1.77 (1.16–2.70)	<0.01
**Platelets (cells/µl)**				
<150,000	0.88 (0.37–2.02)		0.83 (0.36–1.91)	
150,000–400,000	1		1	
>400,000	1.52 (0.98–2.34)	0.12	1.48 (0.98–2.25)	0.17

*Additionally controlled for marital status, education status, monthly income, daily expenditure on food and serum creatinine, none of which were significantly associated with delayed sputum smear conversion.

We found no modification of the relation between anemia and delayed sputum smear conversion by age, sex, mean corpuscular volume (MCV), cigarette smoking, alcohol use, substance abuse, duration of illness, platelets, or BMI (data not shown).

## Discussion

Infection control in TB therapy entails early diagnosis and effective treatment of TB patients. In this regard, sputum smear microscopy at two months of anti TB therapy is key in the assessment of TB therapy outcome. Our current study is one of the few studies that have examined the association of anemia with sputum smear conversion after two months of treatment. In this study, we report two important findings. First, anemia is independently associated with delayed sputum smear conversion at two months of anti TB therapy. Secondly, the relationship between anemia and delayed sputum conversion displays a dose-response effect.

Our findings contradict an earlier study done in similar setting [17] which did not find any association between anemia and sputum culture conversion at one month of anti TB therapy [17]. There are three possible reasons for this difference. First, the smaller sample size in the prior study compared to ours would limit their power to detect small difference if any. Second, the study involved patients with less severe forms of anemia, (all participants had hemoglobin >7 g/dl) [17]. Indeed, as shown in our study, those with milder forms of anemia had a smaller risk for delayed sputum conversion, suggesting the need for a bigger sample size to have adequate statistical power to detect the smaller effect. Third, the discrepancy could be accounted for by difference in methods of outcome assessment: the former study used culture conversion, which is a known more stringent method compared to sputum smear microscopy adopted by our study. Morris et al [22], showed that sputum non conversion among TB patients was correlated with persistency of anemia during treatment of TB. Further, these authors reported lower mean hemoglobin among patients with positive sputum smear compared to those with negative sputum smear at three months.

Mechanisms that explain the association between delayed sputum conversion and baseline anemia are yet to be established. Tuberculosis causes anemia but why some patients do not become anemic is unclear. In previous reports, malnourished TB patients had lower mean levels of hemoglobin and zinc, compared to healthy controls, malnourished controls and well-nourished TB patients [21]. In addition, TB patients with zinc deficiency had impaired T-cell immunity [33]. In our study, underweight (BMI <18.5 kg/m^2^) observed in more than half of the study population, although not directly associated with sputum conversion, could have mediated the complex immune interaction between sputum conversion, anemia, and malnutrition, which can't be determined by the current study. Another possible mechanism for the relationship between anemia and delayed smear sputum conversion could be iron deficiency. Low ferritin levels have been reported to predict treatment failure at one month of TB therapy [34]. Iron deficiency among patients impairs host T-cell immunity mediated through interference with effector cell activity [35], [36].

Evidence on the association between cigarette smoking and sputum conversion is limited and inconclusive [37]. In line with previous studies, we found an increased risk of delayed sputum conversion among current smokers [12], but not among those who had quit smoking. Visser et al [38] did not find a significant association between baseline smoking and sputum culture conversion. This inconsistency may have been caused by different definitions of smoking used in the studies: ‘ever vs never smokers’ (Visser) ‘current vs past vs never smokers’ (our study). Our study found no effect on sputum conversion among past smokers. This finding, however, should be confirmed by other independent studies. Smoking in known to promote mycobacterial adherence to airway epithelial cells by affecting virulence changes in the respiratory tree that favor TB infection and delays clearance of the infection [39].

Older age has been associated with poorer TB outcomes including sputum conversion and death [12], [40]. In our study, we observed a trend of increasing age related to higher risk of sputum conversion. Similar to previous studies, we found that resistance to isoniazid and rifampicin predicted delayed sputum conversion [6], [7]. Patients with mycobacteria TB resistance to isoniazid and rifampicin do not respond well to first-line anti-TB drugs, warranting switch to second-line anti TB. This finding emphasizes the need for drug susceptibility testing (DST) among newly diagnosed TB patients using available GenExpert machines. We found a significant association between lymphopenia and delayed sputum conversion. Both lymphocytosis and lymphopenia have been found to occur among TB patients [22]. Low lymphocyte count has been shown to occur among patients with more severe forms of TB [41] which may in part explain delayed sputum conversion [12]. In agreement with previous studies during the antiretroviral therapy (ART) era, we did not find any significant association between delayed sputum conversion and HIV infection [42], [43]. The lack of association between HIV and delayed sputum conversion is probably indirect evidence for the beneficial effects of the scale up of ART in Dar es Salaam, as well as improved TB/HIV collaborating services demonstrated in settings similar to that of the study area [44]. The outcome of TB patients has been found to be similar regardless of HIV status as long as HIV patients are kept on ART [43]. We did not collect information on ART use for our study, however, in our study setting, all newly identified HIV co-infected TB patients are initiated on ART in the same clinics [45].

Our study had some limitations. First, we excluded 31% of patients for whom sputum smear results at two months was not available or did not have baseline hemoglobin results. In the comparative analysis we found that the excluded patients had, on average, lower lymphocyte counts and were more likely to have had a current or past history of substance abuse. The literature, as well as our current findings suggest that delayed sputum conversion is associated with low lymphocyte count and substance use [12], [41], [43]. It is likely in this scenario that the association between sputum conversion and anemia could be stronger. Second, because we did not perform mycobacteria cultures at two months, there could have been a possibility of reporting non-viable mycobacteria [10]. However, in most cases, smear conversion precedes culture conversion, and almost all smear positive cases are culture positive at two months [6]–[8]. Third, adherence to anti TB is an important factor for sputum conversion and cure and could have introduced bias in our study. In the study setting, anti-TB therapy is administered as daily direct observed therapy (DOT) throughout the duration of treatment by either a health care worker at health facility (facility DOT) or a community treatment supporter (community DOT) according to patient's preference [26]. Adherence to anti-TB in Tanzania has been shown to be as high as 99% when urine samples were tested for metabolites of the drugs among TB patients [46]. Finally, Pregnancy is known to cause anemia, in this study we did not exclude or identify pregnant women. It is worth noting that women composed one- third of the study population.

Despite the limitations, our study has some important contribution to science and policy with regards to patient management. This work is one of the very few current attempts to show the importance of anemia on TB patient outcomes in the era of HIV. It is large and was conducted in a TB programmatic setting making it easier for planning and adoption of strategies and policies that may improve TB patients' outcomes. Given the large burden of anemia among TB patients, representation of the categories of severity of anemia in our study population provides room for priority setting. The dose-response effect of anemia that was shown in this study underscores the rigorousness of the association between anemia and sputum smear conversion. If our findings are taken into consideration, and lead to treatment of anemia among TB patients, there may be decreased infection transmission, as well as decreased mortality associated with TB.

In conclusion, anemia at the initiation TB treatment is significantly associated with delayed sputum smear conversion among sputum positive TB patients with a dose-response effect. The mechanisms mediating this relationship need to be determined in-order to institute effective interventions. Further studies should seek to understand the mechanisms for anemia among TB patients and assess the efficacy of treating anemia in a randomized setting as a means to improve TB outcomes.
